# Lights4Violence: a quasi-experimental educational intervention in six European countries to promote positive relationships among adolescents

**DOI:** 10.1186/s12889-019-6726-0

**Published:** 2019-04-08

**Authors:** C. Vives-Cases, M. C. Davo-Blanes, R. Ferrer-Cascales, B. Sanz-Barbero, N. Albaladejo-Blázquez, M. Sánchez-San Segundo, M. Lillo-Crespo, N. Bowes, S. Neves, V. Mocanu, E. M. Carausu, J. Pyżalski, M. J. Forjaz, I. Chmura-Rutkowska, C. P. Vieira, C. Corradi

**Affiliations:** 1 Faculty of Health Science, University of Alicante, Alicante, Spain; 2 CIBER of Epidemiology and Public Health (CIBERESP), Madrid, Spain; 3 Instituto de Salud Carlos III, Madrid, Spain; 4 Cardiff Metropolitan University, Cardiff, UK; 5 Instituto Universitário da Maia / Maiêutica Cooperativa de Ensino Superior CRL, Maia, Portugal; 6 Universitatea de Medicina si Farmacie Grigore T. Popa, Iasi, Romania; 7 Adam Mickiewicz University, Poznan, Poland; 8 Universidade Aberta – Delegação do Porto, Lisbon, Portugal; 9 REDISSEC, Madrid, Spain; 10 Libera Universita Maria SS Assunta Di Roma, Rome, Italy

## Abstract

**Background:**

Preventing intimate partner violence or dating violence (DV) among adolescents is a public health priority due to its magnitude and damaging short and long-term consequences for adolescent and adult health. In our study protocol, we complement prior experiences in DV prevention by promoting protective factors (or assets) against gender violence such as communication skills, empathy and problem-solving capability through “Cinema Voice”, a participatory educational intervention based on adolescents’ strengths to tackle DV.

**Methods/design:**

A longitudinal quasi-experimental educational intervention addressed to boys and girls ages 13–17 years, enrolled in secondary education schools in Alicante (Spain), Rome (Italy), Cardiff (UK), Iasi (Romania), Poznan (Poland) and Matosinhos (Portugal). Both process and results evaluations will be carried out with 100–120 intervention and 120–150 control group students per city at three time periods: before, after and 6 months after the implementation of the following interventions: 1) Training seminar with teachers to promote knowledge and skills on the core issues of intervention; 2) Workshops with intervention groups, where participants produce their own digital content presenting their perspective on DV; and 3) Short film exhibitions with participants, their families, authorities and other stakeholders with the objective of share the results and engage the community. Outcome measures are self-perceived social support, machismo, sexism, tolerance towards gender violence, social problem-solving and assertiveness as well as involvement in bullying/cyberbullying. Other socio-demographic, attitudes and violence-related co-variables were also included.

**Discussion:**

This study may provide relevant information about the effectiveness of educational interventions that combine a positive youth development framework with educational awareness about the importance of achieving gender equality and preventing and combating gender violence. To our knowledge, this is the first study that involves six European countries in an educational intervention to promote violence protective assets among enrolled adolescents in secondary schools. This study may provide the needed tools to replicate the experience in other contexts and other countries.

**Trial registration:**

Clinicaltrials.gov: NCT03411564. Unique Protocol ID: 776905. Date registered: 18-01-2018.

## Background

There is a growing concern about how intimate partner violence (IPV) is increasingly appearing at earlier ages. In 9 of the 14 areas included in the WHO Multi-Country Study on Women’s Health and Domestic Violence against Women, current physical and/or sexual IPV in the last 12 months among 15 to 24-year-old ever-partnered women was over 30%, with higher prevalence rates than those registered among women aged 25 years and older in most of the studied countries [1]. According to European Union Agency for Fundamental Rights (FRA), out of all women in an intimate partner relationship, 22% have experienced physical and/or sexual violence since the age of 15 [2].

Studies conducted in the United States, where the majority of the studies on this form of gender-based violence are carried out, showed that 20.9% of students had experienced some form of physical and/or sexual dating violence (DV), which is defined as the perpetration of violence by at least one member of an unmarried couple on the other member within the context of dating or courtship [3]. When studies include psychological violence, the prevalence of this type of gender-based violence increases to 65% [4, 5]. DV can also take forms of cyber-violence conducted by computer mediated communication tools. It has been estimated that.17% of the cyberbullying perpetrators targeted a former boyfriend/girlfriend [6, 7].

In addition, DV has been associated with an increase in other violence-related behaviors, including substance use, depression, suicidal behavior, poorer educational outcomes, post-traumatic stress, unhealthy weight control and risky sexual behavior [8]. Moreover, DV can be a precursor to IPV throughout the adult union later in life [9].

In case of gender-based violence or DV among adolescents, we undoubtedly deal with a hidden problem and “gray zone” of youth reality. Due to adults’ ignorance, gender stereotypes and turning a blind eye we know little about forms, consequences and coping strategies adopted by teenagers who experience gender-based violence although such knowledge is necessary for us to be able to provide care and effective help and assistance to silent victims. The research suggests that teenagers’ silence over the problems of gender-based violence is the answer to attitudes presented by adults and the cultural climate surrounding gender identity, gender roles and sexuality [10–12]. Many parents are often unaware of the problem and they hardly ever discuss it with their children. Many members of societies, still believes in many myths about “natural” male and female features, needs and sexual behaviors. What is more, many forms of gender-based violence are considered an inherent element of a man-woman or boy-girl relation. This combination of factors; adults turning gender and sexuality issues in teenage relations into taboo subjects, the lack of training and support for professionals in the schooling systems to competently address these issues, absence of sexual education, highly internalized gender stereotypes and prejudice cause victims to be ashamed and fear stigmatization. Thus, they rarely share their problems with adults or seek help [13–17].

Considering the complexity of gender violence, since the 1990’s, actions have been taken at a European level to eliminate any form of violence against women, including IPV and DV. The European Parliament, the European Council and the European Commission have adopted resolutions, conclusions and strategies about violence against women in general (as a violation of human rights), as well as specific types of violence, such as stalking, honor crimes, and female genital mutilation, among others [18, 19]. The Council of Europe Convention on Preventing and Combating Violence against Women and Domestic Violence is one of the more recent and significant treaty aiming to create a legal framework at pan-European level to protect women against all forms of violence, and prevent, prosecute and eliminate violence against women and domestic violence (CE, 2011) [20].

However, there is still a lack of comparative cross-national studies that assess strategies and interventions related to this issue in different European Member States and that evaluate their relative effectiveness in preventing violence among vulnerable populations such as adolescents.

### Tackling dating violence and promoting positive relationships

Public health research and interventions have begun to focus on positive health, in which actions in health look to so called “health assets” or what individuals, families and communities can do to increase their level of control over and improvement of their health [21]. This model of positive health emphasizes the origins of good health and has become a point of departure for the development of health promotion interventions [22–24].

Programs have been carried out in both the educational and social contexts that focus both on primary prevention (directed at the whole population) and secondary prevention (directed at youth at risk, such as children of women affected by gender violence) focused on gender violence in young people and adolescents. These programs focus on training on incidence and prevalence of violence, myths, power, traditional gender roles, and resources available for victims and perpetrators [25, 26]. Evaluations of these interventions show significant changes in the risks of physical, psychological and sexual violence in both boys and girls [27–30].

The program proposed in this project shares some of the above-mentioned elements, but its objectives relate to the promotion of protective factors (or assets) to prevent gender violence. These assets are related to the capacity for communication, empathy, pro-social abilities, anger management, perspective taking and non-violent conflict resolution [31]. In this sense, it uses a model for positive youth development, centered on individual, family and community efforts to improve and gain control over health [32]. The model emphasizes youth strengths, stressing the development of capacities (personal, moral, cognitive, conceptual and social) that support young people in resisting risk factors, and reducing or confronting behavior problems such as drug use, risky sexual relationships, antisocial behaviors and depression problems [33]. These capacities are related to resources and assets that provide the necessary support and experiences to avoid and deal with risky situations, or to reduce their severity or consequences when they take place [34].

To our knowledge, there are no studies that evaluate dating violence prevention programs aimed at promoting protective assets in young people with the focus on positive youth development. The results obtained in the programs focused on youth violence and bullying are promising [35]. Active participation and the use of tools that appeal to young people, such as video and short films, are also characteristics that help address health assets, given their role in motivating young people to adopt a more active role in carrying out the program activities [36].

This paper describes the study protocol that our team will use to implement and evaluate the *Lights4Violence* project [37], a research action funded by the European Commission Directorate-General for Justice and Consumers Rights, Equality and Citizen Violence Against Women Program 2016, under the grant agreement number 776905, for the period 2017–2019.

## Methods

### Objectives

The overall goal of this study is to contribute to evidence-based strategies to prevent dating violence focused on adolescents’ strengths and capabilities to develop positive relationships with their peers rather than gender violence risk factors. Our objective is to implement and evaluate the effectiveness of an educational intervention, titled “Filming Together to See Ourselves in a New Present”*,* to promote dating violence protective assets among secondary school students from different European cities (Alicante, Rome, Iasi, Matosinhos, Poznan and Cardiff). More specifically, it aims to:

1. Enable adolescents to acknowledge IPV-related protective factors that are present in themselves, their families, the school and other closed settings, and to know how to properly use them;
2. Contribute to education and awareness-raising about the importance of positive interpersonal relationships based on esteem and trust;
3. Support adolescents in challenging sexist and tolerant attitudes towards gender-based violence and dating violence;
4. Promote skills for management of problems and conflicts through interpersonal communication, mediation and negotiation among youth; and,
5. Empower young people to claim their rights and those of their peers to be held in esteem and to protect themselves from at-risk or abusive relationships.

In order to respond to these aims, we will also work with secondary school teachers and engage them with research teams in the implementation of the activities and the core interventions of the project.

### Study design

The study is a longitudinal quasi-experimental educational intervention with a quantitative evaluation. The evaluation will be carried out using an on-line questionnaire distributed to the intervention and the control groups at three time periods: before starting the program (baseline), after finishing the study - including the dissemination phase - (time 1 or T1) and six months after implementation (time 2 or T2). A process evaluation will also be carried out with intervention groups.

### Sample size calculation

A statistical power analysis was performed for sample size estimation, based on data from a previous published random-effects meta-analysis of 23 studies about school-based interventions aimed to prevent violence and negative attitudes in teen dating relationships [38]. The effect sizes (ES) in this meta-analysis for teen dating violence attitudes was g = 0.14, 95% CI (0.10, 0.19), indicating a significant effect size estimation according to Cohen (1998) criteria [39]. With an alpha = .05 and power = 0.90, the projected sample size needed for this effect size, according to G*Power v. 3.1.9.2, was approximately *n* = 430 to detect statistical differences between intervention and control groups in our study. We finally proposed total sample size ranking from 600 to 700 (by each, control and intervention group) to ensure the minimum of 450 in the post-intervention evaluations.

### Participants’ recruitment

Two groups of students will be assigned either an intervention or control condition, respectively. The intervention group will be composed of 100–120 boys and girls aged between 13 and 16–17 years, studying in secondary schools in each targeted city. The control group will be composed of 120–150 students by city from other schools with similar socioeconomic characteristics (relating to social characteristics and school location).

The selection of schools will be carried out by contacting different secondary education centers from the city as considered appropriate by the members of the research team (non-random sample). The program contents will be presented, and the possibility to participate will be offered. The intervention group will be selected from among the students whose schools accept participation in the study (total sample about 750 students). The control group will be made up of students from schools with social characteristics like those that will implement the intervention. The students from the control and the intervention groups will have the same composition in terms of age, sex and academic course. Control and intervention groups will belong to different educational centers in order to avoid contamination. This is a wait-list control study, that is, those schools that participate in the project as a part of the control group will be offered the possibility of participating in teacher training, access to guides and manuals generated by the project and may be able to carry out the intervention in the future (i.e., when the intervention is finished in the intervention group) with our support.

### Lights4Violence Core intervention through “cinema voice”

The core intervention will be developed in five modules, mainly addressed to students except for the two firsts, which will also include secondary school teachers. These five modules comprise between 15 and 17 sessions of approximately 50 min. They will be distributed in ordinary class schedules, involving the teaching staff who has participated in the aforementioned training seminar. See Tables 1 and 2 for more details about sessions.

**Table 1:** Overview of Lights4Violence intervention modules 1 and 2

MODULE 1: ASSETS FOR POSITIVE ADOLESCENT DEVELOPMENT AND THE PROMOTION OF HEALTHY COUPLE RELATIONSHIPS		
SESSIONS	ACTIVITIES	TIME/ACTIVITY (in minutes)
Session 1: Assets for positive adolescent development	Presentation	10
	Identifying health and wellbeing in images	15
	Understanding assets for development: what and where are they?	15
	Linking family, school and community assets with personal assets	20
Session 2: Building a positive common language	Presentation	10
	Phase 1: Reading and adding text to illustrations	20
	Phase 2: Group discussion	30
Session 3: Identifying assets that promote healthy couple relationships	Presentation	10
	Phase 1: Reading and activity 1	10
	Phase 2: Group work activity 2	20
	Phase 3: Group discussion	20
MODULE 2: COMPETENCES THAT PROMOTE HEALTHY COUPLE RELATIONSHIPS		
Session 1: Rebunking myths and irrational beliefs	Presentation	10
	Myth or reality?	15
	Rebunking false beliefs	20
	Ten characteristics of healthy people	15
Session 2: Anger, self control and problem resolution	Presentation	10
	Wrinkled paper technique for working anger	10
	Relaxation technique for working with self-control	15
	BROEV technique for problem solution	15
Session 3: Social skills, assertiveness and self-esteem	Presentation	10
	Communication styles and empathy	15
	Assertiveness: the sandwich technique	20
	Strengthening self-esteem	15
Session 4: Creating Stories about positive couple relationships	Presentation	10
	Creating stories about positive couple relationships	25
	Sharing our stories	20

**Table 2:** Overview of Lights4Violence intervention modules 3, 4 and 5

MODULE 3: PRE-PRODUCTION OF SHORT FILMS		
SESSIONS	ACTIVITIES	TIME/ACTIVITY (in minutes)
Session 1: Constructing creative ideas about healthy couple relationships	Clarifying ideas	10
	Learning to synthesize ideas	15
	Brainstorming	30
Session 2: From idea to plot	Learning about the plot	10
	In search of the SW and 1H	15
	Refining and transforming ideas into a plot	30
Session 3: The final Plot!	Sharing Our stories	55
Session 4: From plot to literary and technical script	Before beginning… we need to know	15
	Practicing the development of a literary and technical script	20
	What shot do we choose?	20
Session 5: The final script	Our script	40
	What does what	15
MODULE 4: PRODUCTION 3, 2, 1… ACTION!		
Session 1: Getting ready	Scenography and general rehearsal	110
Session 2: Filming	Silence, camera and…action!	110–165
MODULE 5: POST-PRODUCTION		
Session 1: Deciding about the assemblage of our video capsules	What story do you want to tell?	55
	Viewing and sharing our short film	55

In addition, we planned to organize two types of dissemination activities. Firstly, a short film exhibition with the support of the city hall and other public institutions. The objective is to provide a space where participants can voluntary present their video capsules and briefly explain the production process. After the students’ brief presentations, the short films will be shown. The public attendees of the event (professors of the schools of the city and province, other students, participants and non-participants, family members, authorities and others involved in the fight against gender violence) will have the opportunity to become familiar with the project and get to know the protective assets of DV that were identified by participants and put into the scenes of the video capsules and short films. All participants will receive a certificate and a prize as acknowledgement of their active participation in the project. And, secondly, the development of teaching guides for the use of resulting short films in other professors’ classrooms. This second activity is designed to facilitate the use of the resulting material by secondary professors that want to address topics related to equality and personal respect in couple relationships in their classes.

### Implementation plan

This study is expected to last 24 months (from December 2017 to December 2019). Its implementation integrates three parts with activities related to the core intervention of the project, evaluation and communication and dissemination. They are further described and scheduled in Table 3.

**Table 3:** Project “Lights4Violence” Implementation planning in months from December 2017 to December 2019

Month number	1	2	3	4	5	6	7	8	9	10	11	12	13	14	15	16	17	18	19	20	21	22	23	24
Core Interventions of the project																								
1.Preparatory tasks		X	X	X	X	X																		
2.Seminar “Promoting Violence Protective Assets together”							X	X																
3.Workshop “Filming Together to see Ourselves in a New Present”									X	X	X	X	X	X	X	X	X	X						
4.Short film exhibitions																			X	X				
5.Short films teaching guide																			X	X	X	X	X	
Project Evaluation																								
1.Action plan evaluation	X	X																						
2.Recruitment of participants							X	X	X															
3.Selection battery tests	X	X	X																					
4.Computer-based evaluation tool			X	X	X	X																		
5.Pilot testing									X	X	X													
6.Data evaluation recruitment & analyses											X	X	X	X	X	X	X	X	X	X	X	X	X	X
Communication and Dissemination																								
1.Communication & dissemination plan	X	X	X																					
2.Web sites	X	X	X	X	X	X	X	X	X	X	X	X	X	X	X	X	X	X	X	X	X	X	X	X
3.Social media				X	X	X	X	X	X	X	X	X	X	X	X	X	X	X	X	X	X	X	X	X
4.Journals						X	X	X	X	X	X	X	X	X	X	X	X	X	X	X	X	X	X	X
5.Newsletters, professional magazines				X	X	X	X	X	X	X	X	X	X	X	X	X	X	X	X	X	X	X	X	X
6.National and international conferences																	X	X	X	X	X	X	X	X

### Evaluation design

Three types of evaluation are planned: formative, results and process evaluation.

Formative Evaluation: Prior to the implementation of the program in the whole sample, an evaluation pilot study will be carried out with a minimum of 20 students (10 boys and 10 girls) per country who are finishing the same grade as those who will later receive the intervention. This pilot study aims: 1) to determine the competencies and capacities of the participants to carry out the on-line questionnaire; 2) to measure the time it takes the students to complete the questionnaire; and, 3) to carry out an internal validation of the questionnaire among the study population by calculating internal consistency and validity indices. The obtained results will be shared among the members of the consortium in order to evaluate possible adaptations considered necessary.

Results Evaluation: A results evaluation will be applied to all students, both in the intervention and control groups at the same time. An on-line questionnaire will be developed in order to evaluate the program results. This will take place in a technology classroom during two classes, preferably before and after the morning break, with a maximum duration of an hour and 15 minutes. For the purposes of the evaluation, the dependent variables will be collected from the following scales:

Student Social Support Scale- Assesses the student’s perceived emotional, appraisal, informational, and instrumental social support received from teachers, parents, close friends, and peers. Students rate each behavior on two dimensions: availability (6-point rating scale) and importance (3-point rating scale) [40].

Questionnaire for Evaluating School Social Climate, Factor 1 - This is a questionnaire that assesses school social climate. It displays a stable factorial structure in two social climate factors: 1) relative to the school and 2) relative to the teaching staff. In this project we will use factor 1 only. The eight items that saturate the first factor are indicative of the capacity for assistance, respect, safety and comfort, as perceived in the school center. Items are rated on a 5-point Likert-type scale, from strongly agree to strongly disagree [41].

Maudsley Violence Questionnaire- Measures a range of cognition relating to violent behavior drawn from clinical and theoretical perspectives. This measure integrates justification of violence in response to threatened self-esteem and the legitimization of violence as central elements. Participants are asked to rate a series of statements as “true” or “false”. The scale is comprised of two factors: ‘machismo’ (42 items) and ‘acceptance of violence’ (14 items) [42].

The Ambivalent Sexism Inventory - A 22-item self-reported measure of sexism. Respondents indicate their level of agreement, on a 6-point Likert-type scale, with various statements. It is composed of two sub-scales whose items may be independently added for sub-scale scores or may be averaged for an overall composite sexism score. The first sub-scale is the hostile sexism scale, which is composed of 11 items designed to assess an individual’s position on the dimensions of dominative paternalism, competitive gender differentiation, and heterosexual hostility, as previously defined. The second sub-scale is the benevolent sexism scale, which is composed of 11 items that aim to assess an individual’s position on the dimensions of protective paternalism, complementary gender differentiation, and heterosexual intimacy, as previously defined [43].

Social Problem-Solving Inventory-Revised Scale- A brief scale of 25 items that measures young people’s ability to resolve their social problems. Items are answered in a 5-point Likert-type scale, ranging from “this is not true” to “extremely true”. Items are distributed in five sub-scales (5 items in each subscale) that evaluate functional and dysfunctional aspects of the ability to problem solve. The functional dimension is evaluated through two sub-scales: Positive Problem Orientation and Rational Problem Resolution; while the dysfunctional dimension is evaluated through the sub-scales Negative Problem Orientation, Avoidance Style and Impulsivity/Carelessness Style. These five dimensions allow obtaining a total score that corresponds to a general estimation of the ability to solve problems, in addition to the average scores in each dimension [44].

Aggression Questionnaire-Refined- Measures four aspects of aggression: Physical Aggression and Verbal Aggression, which involve hurting or harming others and represent the instrumental or motor component of behavior; Hostility, which consists of feelings of ill-will and injustice and represents the cognitive component of behavior; and Anger, which involves physiological arousal and preparation for aggression and represents the emotional or affective component of behavior. It will use a brief version of 12 items which are scored in a five option Likert-type scale ranging from 1 (never) to 5 (always) [45].

Rosenberg Self-Esteem Scale- A 10-item scale that measures global self-worth by assessing both positive and negative feelings about the self. The scale is believed to be unidimensional. All items are answered using a 4-point Likert scale format ranging from “strongly agree” to “strongly disagree” [46].

Assertive Interpersonal Schema Questionnaire- This assertive behavior questionnaire, with 21 items, assesses four dimensions that refer to external emotional support (5 items), practical personal ability (4 items), interpersonal management (8 items) and affective personal ability (4 items). Items are rated on a 1 (completely false) to 5 (completely true) Likert-type scale. Scores on the questionnaire higher than the average in each of the dimensions indicate good personal adjustment and adequate capacity for assertiveness [47].

Subjective Happiness Scale- A global measure of subjective happiness that evaluates wellbeing as a global psychological phenomenon, considering the definition of happiness from the perspective of the respondent. It consists of four items with Likert-type responses with seven options. Scores are the total number of items divided by the sum of the scores obtained [48].

Bullying and cyberbullying scales – adapted from Lodz Electronic Aggression Questionnaire (LEAQ). The tool measure bullying and cyberbullying understood as a serious peer violence that is regular, intentional and involves imbalance of power and includes involvement as a perpetrator and a victim also in the context of involving actual or former romantic partners [49].

In addition, the following co-variables will also be included in the survey:

- Demographic variables – Questionnaire T0: age, sex, birthplace, parents’ birthplace, nuclear family.
- Socioeconomic variables - Questionnaire T0: parents’ employment and parents’ education.
- Violence exposure questions related to [50, 51]:
  - Having (or lacking) a partner
  - Experiences of abuse and/or violence by an adult
  - Exposure to intimate partner violence

For the Process Evaluation, information on the following variables will be collected:

- Percentage of participation in each session, taking the list of students from each group as a reference, and registering the number of students that attend the sessions and activities proposed in the program. This indicator will serve to evaluate program coverage.
- Percentage of hours dedicated to each of the initially foreseen sessions: we will register the number of hours dedicated to each of the described program sessions in each group or class. We will evaluate program completion based on whether the program has been implemented within the time provided or whether more time was needed.
- Evaluation of the participants’ satisfaction with the program. The questionnaire directed to students during the T1 period (at the end of the program) will include the following questions: What are the most important aspects of the program that you would highlight? On a scale ranging from a minimum score of 1 to a maximum score of 10, how would you rate your satisfaction with the program?

### Data analysis

To evaluate the effects of the intervention in terms of the attitudes and behaviors measured in the questionnaires, the change in the response variables will be examined between time 0 (T0) and time 1 (T1), and between T1 and time 2 (T2). A graphic representation of the response variables in T0, T1 and T2 will be produced for both the intervention and control groups. The differences between T0-T1 and T1-T2 will be calculated. Later, in order to quantify the association of the intervention with the change in variables, linear regression models will be constructed for both periods. These models will use, as the main independent variable, the intervention indicator (intervention/control group) as well as the value of the dependent variable in the preceding time period (that is, T0 for the T0-T1 analysis, and T1 for the analysis of the change between T1 and T2).

Later on, multi-level linear regression models will be constructed; level 1 will correspond to the time period (T0, T1 and T2) and level 2 with the group (intervention/control). Using these models, we will analyze the individual change in the response variables of each individual over time (eq. 1) as well as the average trajectory of the group, the variation of the individual trajectories and the magnitude of the change attributable to the intervention, controlling for the co-variables – age, sex, place of origin, socioeconomic level – that could explain the difference between individuals (eq. 2). A significant interaction between the intervention variable (intervention/control) and the time period (T0, T1 and T2) would indicate that the resulting variable is associated differently with the time period in both groups (intervention/control). STATA and SPSS software programs will be used.

For the process evaluation, the success rate of the program implementation will be analyzed, stratified by sex, using the proportion of participants that initiate and finish the program in the three time periods. Finishing the program successfully will be defined as participants having attended at least 80% of training sessions (12/15 sessions) and participated in the assigned program activities. The level of satisfaction with proposed program activities will be evaluated using quantitative measurement of the average score and standard deviation of the variable satisfaction with the program.

### Ethical considerations

All information provided by the project partners and beneficiaries will be confidential. The participation of the target groups will be voluntary and will require a signed informed consent document from the school directors, parents and students. All the project’s procedures and goals will be explained in detail to ensure that potential participants, their parents and teachers are well informed and do not feel forced into giving their consent. Actions will be implemented with professionalism, team work, proximity, availability and flexibility. This project aims to meet the principles of the Convention on the Rights of the Child (art. 19); Helsinki Declaration (AMM, 2013); Convention No. 108 of the Council of Europe of January 28, 1981 for the protection of individuals with regard to the automatic processing of personal data; Directive 95/46 / EC of the European Parliament and of the Council, October 24th Regulation (EU) No 1381/2013 of the European Parliament and of the Council of December 17, 2013 that describes rules for the protection of the rights of persons with disabilities; and Equality and Citizenship for the period 2014 to 2020. If disclosures are made by children during the project that raise concerns about their personal safety or the safety of other children, then the project team will seek to protect the child from further harm and comply directly with child safeguarding legislation within the country in which the research is taking place.

All partners must ask parents and children for their consent to make public the resulting video capsules, short films and photos during project implementation without children’s name attached. They will be asked to provide a signed informed consent to publish and share all these project results for non-commercial purposes and without any kind of modification. They will be assured that the dissemination of these results will be carried out giving the appropriate credit and providing a link to the creative commons license (Attribution & Non-commercial & Non-derivate Creative Commons License: https://creativecommons.org/licenses/by-nc-nd/4.0/).

All institutions and schools participating in the project will be responsible for the care and protection of children. They will be encouraged to adopt codes of good conduct, incorporating the prohibition, prevention and rejection of all forms of violence against children. They will also have the obligation to respect the rights of the child and to report any form of violence or risky situations to competent authorities (directive committee). In addition, the coordinator institution (University of Alicante, Spain) and all partners will ensure that all individuals working in the project in contact with children will have no prior convictions and sanctions and will ensure that everyone will adopt codes of good conduct and good praxis.

## Discussion

We foresee several challenges during the implementation and evaluation of this protocol. The first challenge emerges from the fact that we are going to implement and evaluate the same interventions with teachers and students in six European cities which differ in many aspects, including their prevalence rates of dating violence [52]. Although English is our common language, we must translate and adapt all training materials and selected evaluation indicators into our own languages, so that they can be used with our target populations. This challenge is also one of the added values of the project; it has the future potential to be transferrable to similar European contexts. The second challenge is related to promoting participation of all target groups, both those who will receive the intervention and evaluation and those who will receive only the evaluation, at least at first. In the case of the intervention groups, we consider that the participation of teachers is one of the main drivers of the proposed interventions with children. In the case of the control groups, we will offer the teachers our support and access to all training materials developed during the project so they can implement the workshop with students after the third 6-follow-up evaluation.

As in all longitudinal studies, we may lose cases during the intervention, mainly because the students may move. We think that these losses will affect our sample size very little. If a student does not attend class the day that the class will take the survey, he/she will complete the questionnaire another day. In addition, it is possible that some scales show us that the students have very high capacities. In these cases, it may be difficult to identify changes due to a ceiling effect. Furthermore, effect sizes in psycho-educational interventions are usually small to moderate, and statistical differences may not be found at a country-level due to lack or statistical power. However, this should be overcome when analyzing the total sample.

In relation to the strengths of our study protocol, the interventions proposed in *Lights4Violence* go beyond the transmission of information centered on concepts and actions such as empowerment, in order to endow young people with motivation and learning related to healthy lifestyle habits [53]. They are designed to develop a process of participatory teaching/learning in order to acquire competencies “to know”, “to want” and “to do” in order to achieve a gradual process of empowerment. It is hoped that this process will provide greater control over the decisions and actions that affect how young people relate to determinants of their health and wellness [54]. In addition, the use of participatory techniques and learning resources that we use are especially important to promote adolescents’ involvement in activities and programs. Young people prefer and become more implicated in active projects that include activities such as theatre performances or community activities where they can have “a voice” in reflecting their opinions and ideas [36]. The production of the proposed short films- as a way or reinforcing the previous training in the core concepts and values of *Lights4Violence-* have been recognized as useful tools for learning and for work on health issues with young people and adolescents [55]. Finally, we expect to reproduce the positive results of previous interventions that have also integrated group learning as a pedagogical practice, which have shown the advantages and effectiveness of this type of learning in the integral training of the student [56]. Group learning develops- above all- abilities for social interaction, respect and support that are interrelated with learned knowledge and attitudes. That is to say, it permits integrating the student’s own experiences with the enrichment of the experiences of others. It favors dialogue and active and critical participation related to the topic at hand, and it develops the ability for conflict resolution and working as a group.

To our knowledge, *Lights4Violence* is the first cross-national intervention study to promote positive relationships among adolescents. It is, in fact, the first attempt in our countries to combine previous experiences in preventing DV with the added value of promoting protective factors (or assets) against gender violence related to communication skills, empathy, prosocial affective competencies, anger management, and conflict management without violence. Addressing the current challenge requires new forms of gender violence prevention among those who are involved in their first intimate relationships or even among those who have not been in relationships yet. The adaptation and implementation of this study protocol in primary school students (aged between 11 and 12 years old) would be also a future challenge.

## Abbreviations

DV: Dating Violence
IPV: Intimate Partner Violence
